# Structural Insights into the *Staphylococcus aureus* DltC-Mediated D-Alanine Transfer

**DOI:** 10.3390/biom16010044

**Published:** 2025-12-26

**Authors:** Hanul Jeon, Hyebin Lee, Chiman Song, In-Gyun Lee

**Affiliations:** 1 College of Pharmacy, Research Institute of Pharmaceutical Sciences, Seoul National University, Seoul 08826, Republic of Korea; 2 Chemical and Biological Research Center, Korea Institute of Science and Technology, 5, Hwarang-ro 14-gil, Seongbuk-gu, Seoul 02792, Republic of Korea; 3 Department of Pharmacology, Korea University College of Medicine, 73 Goryeodae-ro, Seongbuk-gu, Seoul 02841, Republic of Korea; 4 Division of Bio-Medical Science and Technology, Korea Institute of Science and Technology (KIST) School, Korea University of Science and Technology (UST), 5, Hwarang-ro 14-gil, Seongbuk-gu, Seoul 02792, Republic of Korea; 5 Natural Products Research Institute, Seoul National University, Seoul 08826, Republic of Korea

**Keywords:** *Staphylococcus aureus*, *dlt* operon, D-alanyl carrier protein DltC

## Abstract

*Staphylococcus aureus* (*S. aureus*) is a major Gram-positive pathogen, and treatment of *S. aureus* infections is often challenging due to widespread antibiotic resistance. In Gram-positive bacteria such as *S. aureus*, D-alanylation of teichoic acids (TA) reduces the net negative charge of the cell envelope and contributes to resistance to diverse antibiotics, particularly cationic antimicrobial peptides. D-alanylation is mediated by the *dlt*ABCD operon, which encodes four proteins (DltA, DltB, DltC, and DltD), all of which is essential for the multistep transfer of D-alanine to teichoic acids. Here, we present the first crystal structure of the *S. aureus* D-alanyl carrier protein DltC and analyze its interaction with DltA using AlphaFold3 and all-atom molecular dynamics simulations. We further show that single substitutions of *Sa*DltA-*Sa*DltC interface residues abolish *Sa*DltC mediated enhancement of *Sa*DltA catalysis. Together, these findings define a catalytically critical *S. aureus* DltA-DltC interface and provide a structural insight for targeting the D-alanylation pathway as a potential anti-*Staphylococcus* strategy.

## 1. Introduction

*Staphylococcus aureus* (*S. aureus*) is a Gram-positive human pathogen that causes a wide spectrum of diseases, from skin and soft tissue infections to life-threatening nosocomial infections [1,2,3]. The global spread of antibiotic-resistant strains—most notably methicillin-resistant *S. aureus* (MRSA) and vancomycin-resistant *S. aureus* (VRSA)—has made the development of new therapeutic strategies urgent [4,5]. One of the key cell envelope mechanisms that contributes to virulence and resistance in Gram-positive bacteria, including *S. aureus*, is D-alanylation of teichoic acids (Tas) [4,5]. This modification attenuates the negative charge of the cell wall, reducing binding of cationic antimicrobial peptides and decreasing susceptibility to several antibiotic classes [6].

Tas are abundant anionic polymers in the Gram-positive cell wall. In *S. aureus*, wall teichoic acid (WTA) is covalently linked to peptidoglycan, whereas lipoteichoic acid (LTA) is anchored at the cytoplasmic membrane interface [6]. D-alanine is incorporated into these Tas by the sequential action of four proteins, DltA, B, C, and D, which are encoded by the *dltABCD* operon [6,7]. Because D-alanylation neutralizes the cell surface charge and modulates antibiotic sensitivity, the Dlt pathway has been considered as an attractive drug target [8,9,10,11,12]. Here, we focus on *S. aureus* DltC, the D-alanyl carrier protein, and its interaction with *Sa*DltA, the D-alanine-D-analyl carrier protein ligase. Among the four Dlt proteins involved in the D-alanylation process, DltA and DltC operate in the cytosol and initiate the process; DltA activates D-alanine using ATP and subsequently transfers the D-alanine adenylate to the conserved serine residue of DltC (Ser36 in *S. aureus* DltC), which is post-translationally modified with 4′-phosphopantetheine (Ppant) cofactor, forming DltC-Ppant-D-alanine. DltC is crucial for the transfer of D-alanine to the cell surface with the aid of DltB and DltD, through a not yet fully understood mechanism [3,13,14,15]. Thus, understanding the interaction between DltA and DltC is critical to comprehending the D-alanylation pathway.

Here, we present the crystal structure of *Sa*DltC and analyze its interface with *Sa*DltA with AlphaFold3 modeling and all-atom molecular dynamics simulations. We further show that single-point substitutions of Ser36 in *Sa*DltC—the Ppant attachment site that accepts D-alanine from DltA—as well as of Asp35 and Phe37, residues that lie at the predicted *Sa*DltA-*Sa*DltC interface, abolish the *Sa*DltC-mediated catalytic enhancement of *Sa*DltA activity. Together, these results define a catalytically essential *S. aureus* DltA-DltC interface and provide structural insight for targeting TA D-alanylation as a potential anti-*Staphylococcus* strategy.

## 2. Materials and Methods

### 2.1. Gene Cloning and Protein Expression, and Purification

The cDNAs encodes *Sa*DltC (UniProt ID: P0A018) was amplified and expressed as His_6_-tagged protein using the pET28a expression vector (Takara Bio, Kusatsu, Japan). The cDNA that encodes *Escherichia coli* (*E. coli*) acyl carrier protein synthase (AcpS, UniProt P24224) was amplified and cloned into the pBAD expression vector (New England Biolabs [NEB], Ipswich, MA, USA), resulting in non-tagged AcpS (for co-expression with wild-type *Sa*DltC). The point mutations were introduced using a QuikChange site-directed mutagenesis kit (Agilent Technologies, Santa Clara, CA, USA). *Sa*DltC^WT^ was co-expressed with AcpS in *E. coli* Rosetta (DE3) cells (Thermo Fisher Scientific, Waltham, MA, USA) to produce Ppant-attached *Sa*DltC. The *E. coli* cells were grown in Luria–Bertani (LB) medium at 37 °C until the OD_600_ reached 0.5, and the protein expression was induced with 0.25 mM IPTG. The bacterial cell culture was further grown at 37 °C for 4 h. The cells were centrifuged at 8000× *g* and resuspended in purification buffer A (50 mM Tris–HCl pH 8.0, 500 mM NaCl). After cell lysis by ultrasonication, cell lysate was centrifuged at 20,000× *g* and bound to an Ni-NTA affinity column. After washing with Buffer A, the bound fractions were eluted with Buffer B (Buffer A supplemented with 300 mM imidazole). The His_6_-tag was removed by the addition of TEV protease overnight. The non-tagged protein was further purified via size-exclusion chromatography using a HiLoad 16/60 Superdex 75 pg column (GE Healthcare, Chicago, IL, USA) equilibrated with 20 mM Tris pH 7.5, 200 mM NaCl. The purified protein was concentrated to 20 mg ml^−1^. *Sa*DltC mutant proteins were also expressed in *E. coli* Rosetta (DE3) and purified essentially identically to wild-type *Sa*DltC. The purity of the proteins was analyzed by SDS-PAGE.

### 2.2. Crystallization and Determination of Crystal Structure

Recombinant *Sa*DltC^WT^ protein at a concentration of 20 mg mL^−1^ in 20 mM Tris, pH 7.5, and 200 mM NaCl was used for initial screening of crystallization conditions. A total of 1 µL of protein solution was mixed with an equal volume of reservoir solution consisting of 30% PEG 400, 0.2 M sodium thiocyanate, 0.2 M magnesium chloride, and 0.1 M HEPES, pH 7.5, at 16 °C. X-ray diffraction data was collected at 100 K using an ADSC Quantum 315r CCD detector system at the BL-7A beamline of Pohang Light Source (Republic of Korea). The S*a*DltC^WT^ crystal belonged to the monoclinic space group P2_1_, with unit-cell parameters a = 33.14 Å, b = 63.97 Å, c = 77.14 Å, α = γ = 90.00°, β = 93.95°. The raw data were processed and scaled using HKL-2000 [16]. *Sa*DltC^S36A^ protein crystal was obtained from 14% PEG 8 K, 18% PEG 400, 0.1 M magnesium chloride, and 0.1 M Tris-HCl pH 7.5 at 16 °C. A set of X-ray diffraction data was collected at 100 K, also at the BL-7A beamline of Pohang Light Source. The *Sa*DltC^S36A^ crystal belonged to the orthorhombic space group P2_1_2_1_2, with unit cell parameters a = 32.73 Å, b = 157.96 Å, c = 27.8 Å, and α = β = γ = 90.00°. The raw data were processed and scaled as described above (Table 1).

### 2.3. Molecular Dynamics

MD simulations of the *Sa*DltA-*Sa*DltC complex were performed with GROMACS 2024.3 [17]. The Alphafold3-predicted *Sa*DltA-*Sa*DltC complex was used as an initial structure [18]. The AMBER99SB-ILDN force field and TIP3P water were employed for protein parameters [19]. Nonbonded interactions were calculated using the Particle Mesh Ewald (PME) method with a 1.0 nm cutoff [20]. Newton’s equations of motion were integrated using the leap-frog algorithm with a 2 fs time step, and all hydrogen bonds were constrained using Linear Constraints Solver (LINCS) algorithm [21]. After energy minimization, the system was equilibrated for 1 ns in the NVT ensemble at 300 K using the V-rescale thermostat with backbone position restraints [22], followed by 2 ns NPT equilibration at 300 K and 1 bar using the Parrinello–Rahman barostat [23]. Production MD simulations of 100 ns was then carried out in the NPT ensemble without restraints. Structural analysis including r.m.s.d., Rg, and inter-chain distances were calculated using GROMACS tools. The inter-chain distance was evaluated as the Euclidean distance between the mass-weighted centers of mass of *Sa*DltA and *Sa*DltC using gmx distance after PBD correction.

### 2.4. Pyrophosphatedetection Assay

The adenylation activity of recombinant *Sa*DltA was assessed using a pyrophosphate detection assay. Reactions were carried out in the presence of 5 μM *Sa*DltA, 5 units ml^−1^ inorganic pyrophosphatase, 5 mM ATP, 100 mM KCl, 10 mM MgCl_2_, 50 mM Tris–HCl (pH 7.4), and 5 mM D-alanine at 37 °C. The reaction was initiated by the adding D-alanine, and 20 μL aliquots were collected at 0, 1, 2, 4, 6, and 10 min. Each aliquot was immediately mixed with 380 μL dye solution containing 0.033% (*w*/*v*) malachite green and 1.3% (*w*/*v*) ammonium molybdate in 1.0 M HCl and incubated for 90 s. Absorbance at 620 nm was measured using a BioSpectrometer (Eppendorf, Hamburg, Germany), and reactions lacking 5 μM *Sa*DltA served as blanks. Determination of the adenylation activity of *Sa*DltA in the presence of 300 μM WT or D35A, S36A, F37A mutant *Sa*DltC was determined under essentially identical conditions. Initial rates of the enzyme reaction were derived from phosphate accumulation over time.

## 3. Results

### 3.1. Crystal Structures of Wild-Type and Ser36Ala Mutant S. aureus DltC

For *Sa*DltC to accept the D-alanine adenylate, Ser36 of *Sa*DltC must be post-translationally modified with a Ppant group by the phosphopantetheinyl transferase AcpS [3]. DltA then transfers the D-alanyl moiety from D-Ala-AMP to the thiol of this Ppant prosthetic group covalently attached to Ser36 of *Sa*DltC, forming a thioester [3]. To understand the structural basis of *Sa*DltC recognition by *Sa*DltA, we determined the crystal structures of the wild-type (*Sa*DltC^WT^) and non-modifiable Ser36Ala mutant (*Sa*DltC^S36A^). Both structures were solved by the molecular replacement using *B. subtilis* DltC [24] as the search model (Table 1). The *Sa*DltC^WT^ contained four DltC molecules in the asymmetric unit (chains A–D), whereas contains *Sa*DltC^S36A^ contains two molecules (chains A and B). Chains within each asymmetric group are essentially identical, with Cα root mean square deviation (r.m.s.d.) values of ~0.14–0.28 Å for *Sa*DltC^WT^ and ~0.24 Å for *Sa*DltC^S36A^, so unless otherwise noted, we describe chain A for both structures.

**Figure 1:**
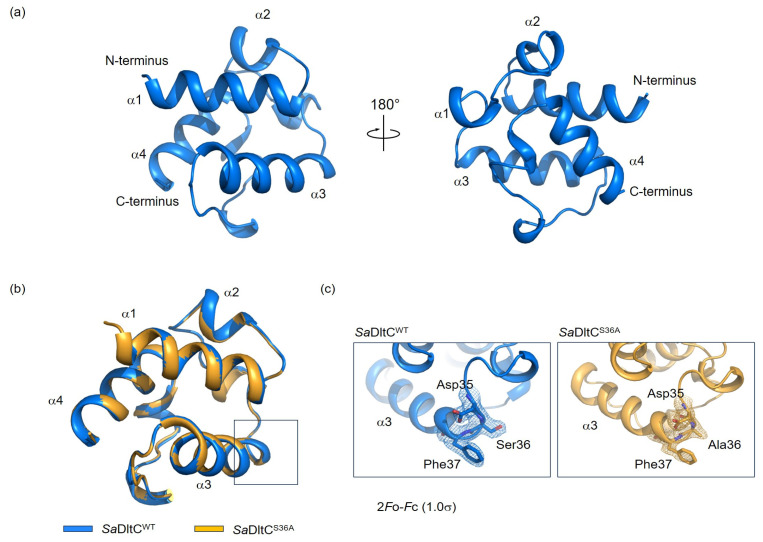
Crystal structures of wild-type *Staphylococcus aureus* DltC (*Sa*DltC^WT^) and the Ser36Ala mutant (*Sa*DltC^S36A^). (**a**) Overall structure of *Sa*DltC^WT^ in ribbon representations. Two orientations related by a 180° rotation are shown. (**b**) Superimposition of *Sa*DltC^WT^ (blue) and *Sa*DltC^S36A^ (orange). The structures are globally similar. Boxes indicate Ser36 residues shown in panel c. (**c**) Close-up views and 2*F*o-*F*c electron density contoured at 1.0σ around the indicated residues for *Sa*DltC^WT^ (**left**) and *Sa*DltC^S36A^ (**right**).

The overall structure of *Sa*DltC^WT^ adopts a compact, globular fold consisting of four α-helices: α1 (residues 2–15), α2 (residues 19–22), α3 (residues 38–50), and α4 (residues 67–75) (Figure 1a). Several DltC structures have been elucidated, including the Ppant-loaded form (PDB ID: 4BPH), the apo (non-Ppant modified) form (PDB ID: 4BPG), and the DltB-complexed structures (PDB ID: 8JF2 and 6BUG) [13,24,25]. In all contexts, DltC adopts a highly similar globular fold, with small overall Cα r.m.s.ds between structures, indicating that its compact, globular architecture is strongly conserved during acceptance and handover of D-alanine (Supplementary Table S1). Also, the *Sa*DltC^Ser36Ala^ structure was highly similar to *Sa*DltC^WT^ with Cα r.m.s.d. of 0.19 Å, indicating that the mutation does not perturb the global fold of the protein (Figure 1b,c).

### 3.2. AF3 Prediction- and MD Simulation-Based Architecture of the SaDltA-SaDltC Interface

Although recent studies have elucidated complex structures of DltC with other Dlt-family proteins, such as DltB and DltD [13,25]—clarifying the architecture of Dlt-mediated D-alanylation and the transfer of D-alanine from the cytosol to TAs—the high-resolution DltA-DltC complex has not been characterized, presumably due to the transient and weak nature of its interaction. *Sa*DltA comprises two structurally distinct lobes, an N-terminal lobe (residues 1–377) and a C-terminal lobe (residues 382–485), linked by a flexible interdomain hinge region (residues 378–381) [26]. Following ATP-dependent adenylation of D-alanine, the C-lobe rotates from the adenylation state to the thiolation state, positioning the D-alanyl-AMP for transfer to DltC [14,15]. We therefore hypothesized that DltC binds DltA in the thiolation state, with the Ppant on Ser36 positioned near the D-alanyl group of D-Ala-AMP.

**Figure 2:**
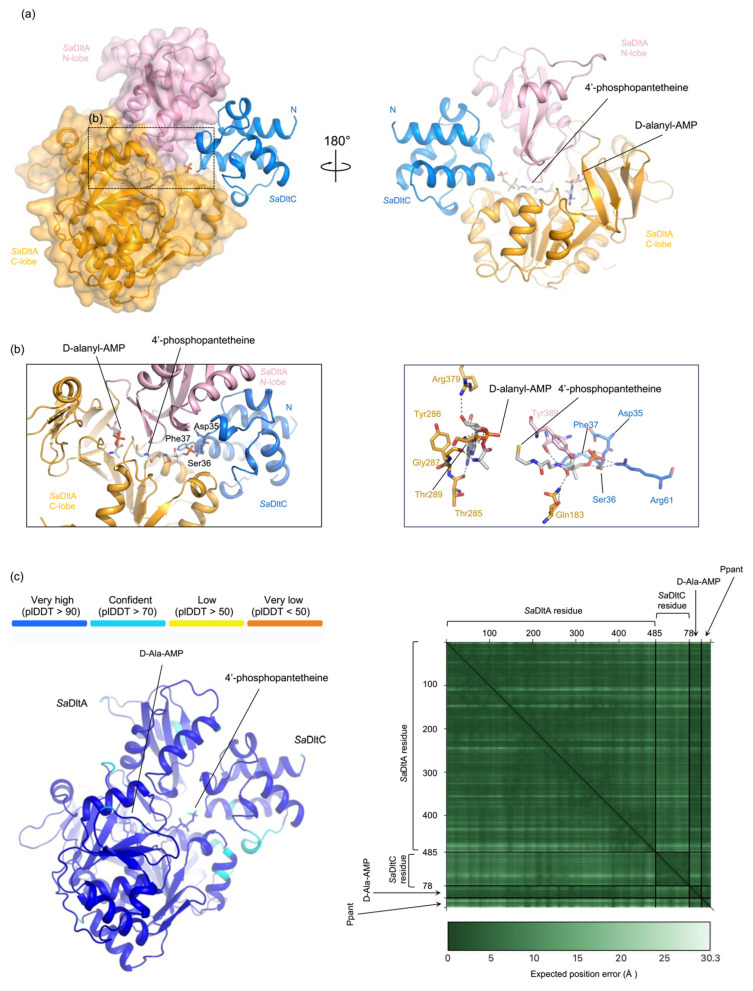
AF3 model of the *Sa*DltA-*Sa*DltC complex with D-alanyl-AMP and 4′-phosphopantethein. (**a**) Two orientations (180°) of the AlphaFold3 (AF3) prediction showing *Sa*DltA (orange) and *Sa*DltC (blue). D-alanyl-AMP and 4′-phosphopantetheine (Ppant) are shown as sticks. *Sa*DltA adopts a thiolation conformation similar to that of *B. subtilis* DltA (PDB ID: 3E7W). (**b**) Close-up views of the *Sa*DltA-*Sa*DltC AF3 model interface. (**c**) (**Left**), per-residue confidence (pLDDT; color scale shown). (**Right**), predicted aligned error (PAE) heat map.

To test this, we used AlphaFold3 (AF3) to predict the complex structure, using *Sa*DltA, *Sa*DltC, D-alanyl-AMP, and 4′-phosphopantetheine as input. The resulting model revealed *Sa*DltA in a thiolation conformation, similar to thiolation conformation of *B. subtilis* DltA (PDB ID: 3E7W) [14], with D-alanyl-AMP bound in the active site (Figure 2a). According to PLIP analysis [27], D-Ala-AMP was located in the active site of *Sa*DltA, making extensive hydrogen bonds and hydrophobic interactions (Figure 2b). Notably, the D-alanyl moiety was positioned adjacent to the thiol group of Ppant, while the phosphate end of the Ppant localizes near Ser36 of *Sa*DltC (Figure 2b). Confidence metrics were high for both proteins (average pLDDT 0.94 for *Sa*DltA; average pLDDT 0.89 for *Sa*DltC) with a low interfacial PAE (~6.0 Å), supporting the reliability of the predicted *Sa*DltA-*Sa*DltC interface (Figure 2c). We also performed an AlphaFold3 prediction of the DltC-DltA complex from *B. subtilis*, and the predicted overall architecture is similar; the Ppant-accepting serine in *B. subtilis* DltC (Ser36) adopts a comparable orientation, positioning its side chain toward the phosphopantetheine phosphate head group (Supplementary Figure S1).

**Figure 3:**
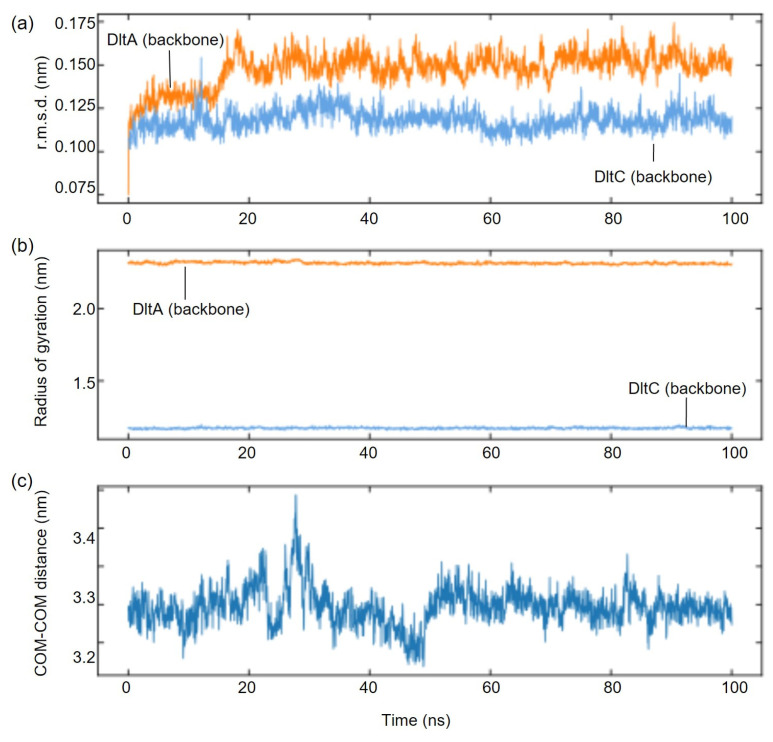
MD analysis for the *Sa*DltA-*Sa*DltC complex. (**a**) Backbone r.m.s.d of *Sa*DltA (orange) and *Sa*DltC (blue) over a 100 ns trajectory. (**b**) Radius of gyration (Rg) of backbone atoms for *Sa*DltA (orange) and *Sa*DltC (blue). (**c**) Center-of-mass (COM-COM) distance between *Sa*DltA and *Sa*DltC as a function of time.

To further assess the interaction between *Sa*DltA and *Sa*DltC, we carried out 100 ns all-atom molecular dynamics (MD) simulations using AF3-predicted complex structure as an initial model (Figure 3). Throughout the trajectory, the complex assembly remained stable and compact, without signs of dissociation. Backbone r.m.s.d. values for each *Sa*DltA and *Sa*DltC proteins were stable (*Sa*DltA, ~1.2–1.8 Å; *Sa*DltC, ~0.7–1.1 Å), radii of gyration were also constant (*Sa*DltA, ~23 Å; *Sa*DltC, ~11 Å), and the inter-chain center-of-mass distance fluctuated narrowly around 32~33 Å (Figure 3). Also, the Ser36 of DltA remained stable during the course of MD simulations (Supplementary Figure S2). Collectively, these AF3-predicted structures, as well as the MD simulation data, support the conclusion that (i) *Sa*DltA in the thiolation state engages DltC and (ii) the *Sa*DltC Ser36-centered interface is geometrically compatible with D-alanyl transfer.

### 3.3. Mutations of SaDltA-SaDltC Interface Residues Abolish SaDltC-Mediated Enhancement of SaDltA Catalysis

**Figure 4:**
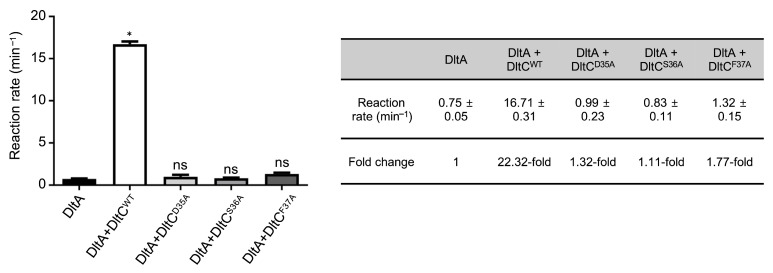
*Sa*DltC-dependent activation of *Sa*DltA. Initial reaction rates for *Sa*DltA alone and with *Sa*DltC variants. *Sa*DltC^WT^ strongly stimulates *Sa*DltA, whereas other mutants show no significant enhancement of the activity of *Sa*DltA. The data are presented as mean ± SD from three independent experiments. Significant differences were determined by Kruskal–Wallis test followed by Dunn’s post hoc test (* *p* < 0.1; ns, not significant).

To test the functional relevance of residues highlighted by the AF3 model and MD simulations, we generated alanine substitution mutants at the interface hotspot (Asp35, Ser36, and Phe37) of *Sa*DltC. Adenylation activity of *Sa*DltA was assessed using pyrophosphate detection assays in the presence of WT or mutant *Sa*DltC. *Sa*DltC^WT^ significantly stimulated *Sa*DltA adenylation activity (22.3-fold increase), whereas the Ser36Ala mutant, lacking the serine required for Ppant modification, showed no activation, consistent with a previous report [26] (Figure 4). In addition to the Ser36Ala mutant, mutations of the neighboring residues (Asp35Ala and Phe37Ala) also abolished the significant stimulatory effect, indicating that a *Sa*DltA-*Sa*DltC interaction interface centered around Ser36 of *Sa*DltC is critical for enzymatic function (Figure 4).

## 4. Discussion

*S. aureus* is a major opportunistic human pathogen that employs diverse immune evasion strategies to cause infections ranging from skin infections to severe, invasive diseases. Further, the emergence of antimicrobial resistance, including methicillin-resistant *S. aureus* (MRSA), makes *S. aureus* notorious and complicates its treatment. Current therapeutic options remain limited, underscoring the urgent need for development of new antibacterial strategies [28]. In this context, we studied the structural and functional interplay between two proteins, *S. aureus* DltA and DltC, components of the Dlt protein family that confer resistance to multiple antibacterials in *S. aureus*. Dlt proteins catalyze the D-alanylation of teichoic acids in Gram-positive bacteria, which reduces net charge and thus promotes resistance to cationic antimicrobial peptides. In this pathway, DltA adenylates the D-alanine to form D-ala-AMP and transfers the D-alanyl moiety to the 4′-phosphopantethein (Ppant) arm attached to Ser36 of *Sa*DltC, the acyl carrier protein. Thus, the DltA-DltC interaction is central to D-alanyl transfer to the cell wall and, consequently, to antimicrobial resistance.

Here, we report two crystal structures of *S. aureus* DltC (wild-type and Ser36Ala mutant). To our knowledge, these represent the first DltC structure from *S. aureus*. Both *Sa*DltC crystal structures—wild-type and Ser36Ala mutant—reveal the similar compact globular fold composed of mainly four alpha-helices. DltA is known to transfer D-alanyl-AMP to DltC, initializing subsequent D-alanine transfer from cytosolic space to TAs in the bacterial cell wall. However, this DltA-DltC interaction is thought to be weak, and no high-resolution complex structure has been available. To characterize the weak, transient *Sa*DltA-*Sa*DltC interaction, we combined AF3 structure prediction with all-atom MD simulations. The AF3-predicted model places the Ser36-linked Ppant arm of *Sa*DltC within reach of the D-alanyl group of D-Ala-AMP bound to the catalytic site of *Sa*DltA, consistent with a thiolation-competent configuration. High confidence metrics of the AF3 prediction supported the model’s reliability. To further characterize the interaction, we performed MD simulations and revealed a stable assembly over 100 ns, with *Sa*DltC Ser36 maintaining interactions at the DltA-DltC interface. Mutation of interface residues impaired *Sa*DltA catalytic activity, further validating this binding mode. In the DltB-DltC complex structure, DltC interacts with DltB with an interface that involves not only the Ppant-attached serine residue but also additional structural elements, including the long α3-α4 loop. Similarly, Ser36 together with adjacent structural elements is likely to contribute to DltA-binding surface, providing additional stability and specificity for productive handover during the D-alanylation cycle. Overall, our study specifically shows the overall plausible architecture of the *Sa*DltA-*Sa*DltC interaction, pinpointing Ser36 for the thiolation and D-alanine transfer reaction. Recently, D-Ala-AMP analogs that inhibit DltA enzymatic activity have been shown to re-sensitize methicillin-resistant *S. aureus* to antibiotics, validating the Dlt pathway as a promising target for novel anti-*staphylococcus* strategy [29]. In this regard, our structural insight may help to design new classes of antibiotics targeting *S. aureus*.

## 5. Conclusions

Our X-ray crystal structures of *Sa*DltC^WT^ and *Sa*DltC^S36A^ revealed that the DltC protein adopts a compact globular fold that is not perturbed by the Ser36Ala mutation, confirming the stability of the core structure. Using an AlphaFold3 prediction and molecular dynamics (MD) simulations, we provided a model architecture for the transient *Sa*DltA–*Sa*DltC complex. Further, the functional relevance of this predicted interface was validated by in vitro biochemical assays. In conclusion, using combined structural and biochemical approaches, we provide insights for the rational design of new antibacterial agents specifically targeting the *dlt* operon-mediated cell envelope modification pathway in antibiotic-resistant *S. aureus*.

## Figures and Tables

**Table 1:** Data collection and refinement statistics.

Data Collection	*Sa*DltC^WT^	*Sa*DltC^S36A^
Wavelength (Å)	0.97927	0.97935
Space group	P 2_1_	P 2_1_ 2_1_ 2
Cell dimensions		
*a*, *b*, *c* (Å)	33.14 63.97 77.14	32.73 157.96 27.80
α, β, γ (°)	90.00 93.95 90.00	90.00 90.00 90.00
Resolution (Å)	32.97–2.28 (2.34–2.28) ^a^	50.00–2.09 (2.14–2.09)
*R*_meas_	0.109 (0.517)	0.216 (1.11)
*R_pim_*	0.041 (0.196)	0.062 (0.333)
*I*/*σ*(*I*)	26.6 (6.77)	18 (3.8)
No. reflections	222,784 (14,764)	222,344 (9149)
No. unique reflections	14,689 (1453)	8957 (891)
Completeness (%)	99.7 (98.6)	99.9 (99.9)
Redundancy	7.2 (6.8)	12.1 (10.2)
Wilson *B*-factor (Å^2^)	29.53	26.60
*CC*_1/2_	0.996 (0.944)	0.997 (0.858)
**Refinement**		
Resolution (Å)	32.97–2.28 (2.36–2.28)	32.05–2.09 (2.17–2.10)
*R*_work_/*R*_free_ (%)	20.0 (20.3)/25.8 (25.5)	21.3 (23.5)/25.9 (26.7)
No. atoms		
Protein	2544	2573
Ligand/Ion	-	1
Water	76	67
*B*-factors (Å^2^)		
Protein	36.4	36.13
Ligand/Ion	-	40.2
Water	37.1	39.3
R.m.s. deviations		
Bond lengths (Å)	0.008	0.007
Bond angles (°)	0.95	0.85
Ramachandran (%)		
Favored		100.00
Outliers	1.39	0.00
PDB code	9XGL	9XGM

^a^ Values in parentheses correspond to the highest-resolution shell.

## Data Availability

The original contributions presented in this study are included in the article/Supplementary Material. Further inquiries can be directed to the corresponding author.

## Supplementary Materials

The following supporting information can be downloaded at https://www.mdpi.com/article/10.3390/biom16010044/s1, Figure S1: Comparison of AF3 predicted DltA-DltC from *S. aureus* and *B. subtilis*. Figure S2: MD analysis for the SaDltC Ser36. Table S1: Structures of DltC homologs.
