# Supplementary material for: Structural Insights into the Staphylococcus aureus DltC-Mediated D-Alanine Transfer
Source: Biomolecules. 2025 Dec 26;16(1):44. doi: 10.3390/biom16010044 (PMC12838833; doi:10.3390/biom16010044)
Supplement: Supplementary file 1 [file biomolecules-16-00044-s001.zip › biomolecules-4023016-supplementary.pdf]

PDB ID	Resolution (Å)	Species	Construct/ complex state	Cα r.m.s.d. with *Sa*DltC	Methodology	Reference
4BPF	1.0	*Bacillus subtilis*	Ser36Ala	0.6	X-ray	(2015) *FEBS Lett* 589: 2283
4BPG	2.2	*Bacillus subtilis*	Wild type	0.8	X-ray	
4BPH	1.8	*Bacillus subtilis*	Wild type (Ppant bound)	0.5	X-ray	
8JF2	3.5	*Staphylococcus thermophilus*	In complex with DltB	2.3	Cryo-EM	(2024) *Nat Commun* 15: 3404
1HQB/1DV5	N/A	*Lacticaseibacillus casei*	Wild type	1.6	NMR	(2001) *Biochemistry* 40: 7964-7972
7R49	1.8	*Lactiplantibacillus plantarum*	In complex with AcpS	1.1	X-ray	(2022) *Sci Rep* 12: 13133
6BUG/6BUH	3.2	*Streptococcus thermophilus*	In complex with DltB	1.2	X-ray	(2018) *Nature* 562: 286-290

Supplementary Table S1. Structures of DltC homologs

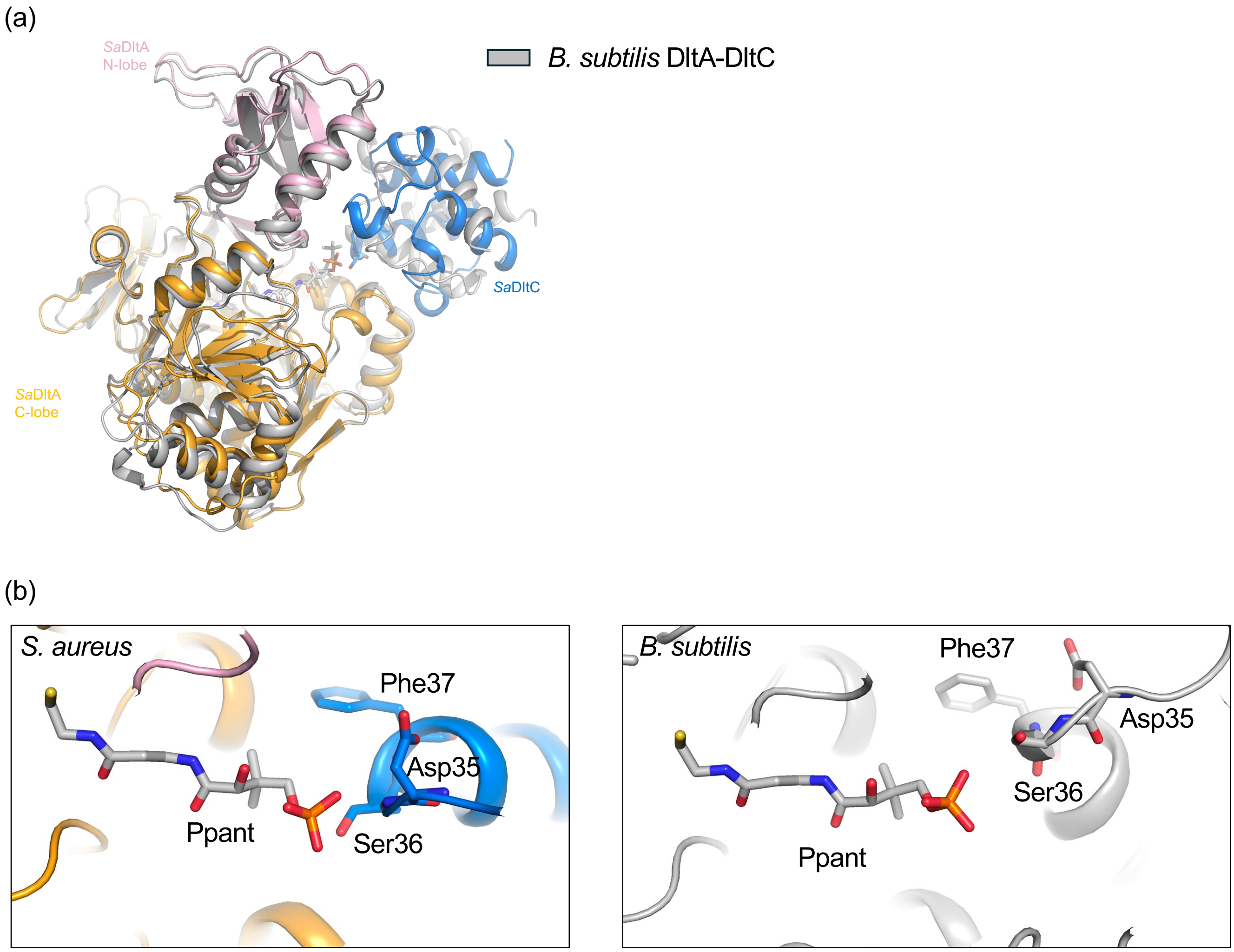

Supplementary Figure S1. Comparison of AF3 predicted DltA-DltC from *S. aureus* and *B. subtilis*. (a) Overall structural comparison of *S. aureus* DltA-DltC complex (pink, orange, and blue) and *B. subtilis* DltA-DltC complex (grey). **(b)** Close-up view of Ppant binding pocket from *S. aureus* (left) and *B. subtilis* (right).

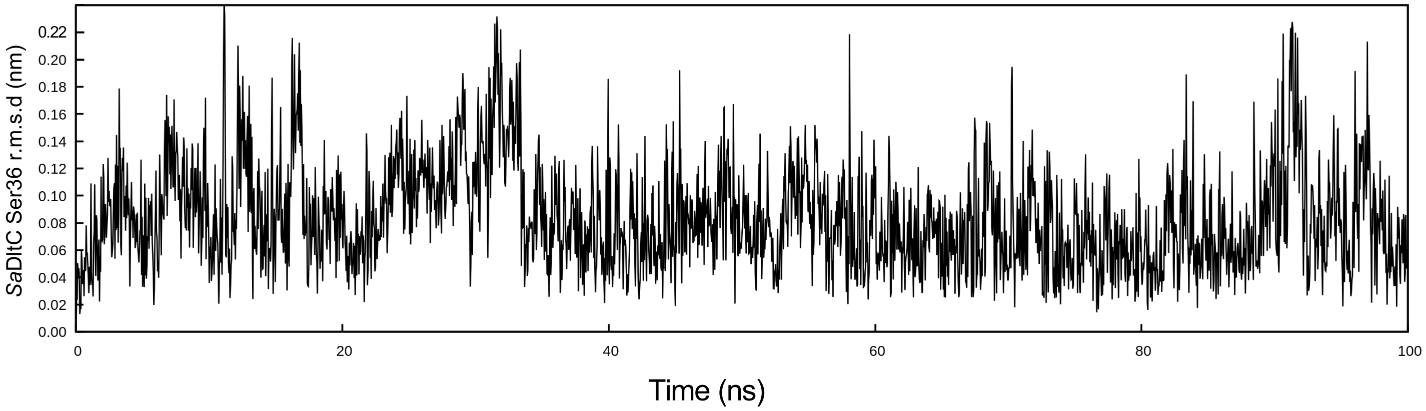

Supplementary Figure S2. MD analysis for the *Sa*DltC Ser36. (a) Backbone r.m.s.d of *Sa*DltC Ser36 over a 100-ns trajectory.
